# When Nausea and Vomiting Are Something Else Entirely, It’s Not Gastroparesis: A Report of Two Cases

**DOI:** 10.7759/cureus.112799

**Published:** 2026-07-16

**Authors:** Ryan Shaw, Keri Allen, Camille Thelin, Rebecca H Klam, Joseph A Sujka

**Affiliations:** 1 Department of Medicine, University of South Florida Morsani College of Medicine, Tampa, USA; 2 Department of Gastroenterology, University of South Florida Morsani College of Medicine, Tampa, USA; 3 Department of Digestive Disease, Tampa General Hospital, Tampa, USA; 4 Department of Gastrointestinal Surgery, University of South Florida Morsani College of Medicine, Tampa, USA

**Keywords:** delayed gastric emptying, gastric volvulus, gastroparesis, mechanical obstruction, motility disorder, roux-en-o configuration

## Abstract

Gastroparesis, a chronic motility disorder, is a diagnosis of exclusion that necessitates careful evaluation for structural and surgically correctable causes. Although the medical differential diagnosis emphasizes functional etiologies, anatomic abnormalities are often under-recognized despite guideline recommendations for thorough endoscopic and structural assessment. Symptoms such as nausea, vomiting, early satiety, and delayed gastric emptying may be indistinguishable from true motility disorders. We present two patients initially diagnosed with gastroparesis whose symptoms were ultimately driven by surgical pathology.

Case 1 describes a 70-year-old woman with persistent nausea and vomiting, weight loss, epigastric abdominal pain, and delayed gastric emptying on scintigraphy, initially scheduled for gastric peroral endoscopic myotomy (G-POEM) for presumed gastroparesis. Intraoperative endoscopy revealed gastric volvulus as the underlying etiology, prompting conversion to definitive surgical repair. Gastric volvulus, while rare, may be complicated by ischemia or perforation, highlighting the importance of timely differentiation from gastroparesis.

Case 2 describes a 53-year-old woman with intractable nausea, vomiting, and inability to tolerate oral intake following a Roux-en-Y gastric bypass (RYGB). Surgical exploration revealed a Roux-en-O configuration with twisted limbs causing impaired drainage, ultimately requiring bypass reversal and pyloroplasty. The Roux-en-O configuration is a rare but serious complication of RYGB involving misplaced anastomoses that create a blind loop, resulting in obstruction and gastroparesis-like symptoms. Clinicians should maintain heightened suspicion for structural pathology in patients with atypical presentations, previous surgery, or refractory symptoms, as delayed diagnosis can lead to life-threatening complications.

## Introduction

Gastroparesis and structural gastric outlet obstructions are fundamentally different pathologic processes and conditions; however, they may present similarly, which can complicate or even delay diagnosis. Gastroparesis is a chronic functional motility disorder characterized by delayed gastric emptying [1]. Gastroparesis can be due to neuropathic processes (i.e., diabetes and post-vagotomy states), idiopathic processes, or myopathic processes [2]. Gastric volvulus is an acute or chronic mechanical obstruction resulting from abnormal rotation of the stomach [3]. In adults (median age of 71-75 years), gastric volvulus is predominantly secondary to paraesophageal hiatal hernias, but may also occur due to diaphragmatic defects, congenital anomalies, or adhesions from previous surgeries [3]. Chronic gastric volvulus can present with various gastrointestinal, abdominal, and chest symptoms, but when acute, gastric volvulus can lead to vascular compromise due to twisting of the stomach [4]. While the risk of acute ischemia is relatively rare, it carries a high mortality rate, emphasizing the need for accurate and timely differentiation from gastroparesis [1].

Beyond naturally occurring structural abnormalities, iatrogenic anatomical alterations following bariatric surgery can also mimic gastroparesis. A history of foregut or bariatric surgery should raise suspicion for underlying structural abnormalities, rather than functional dysmotility alone. The Roux-en-O configuration is a rare but serious surgical misconstruction following Roux-en-Y gastric bypass (RYGB) surgery that presents with nausea, abdominal pain, and chronic bilious vomiting [5-6]. This occurs when the biliopancreatic (BP) limb is erroneously anastomosed to the gastric pouch instead of the Roux (alimentary) limb; with the jejuno-jejunostomy aberrantly positioned, this creates a closed-loop obstruction preventing appropriate drainage [5]. ”Roux-en-O" refers to the circular configuration of the misconnected bowel limbs. This case series underscores how gastroparesis is a diagnosis of exclusion, requiring careful evaluation to rule out structural causes of delayed gastric emptying.

## Case presentation

Case 1: gastric volvulus after hiatal hernia repair

**Figure 1:**
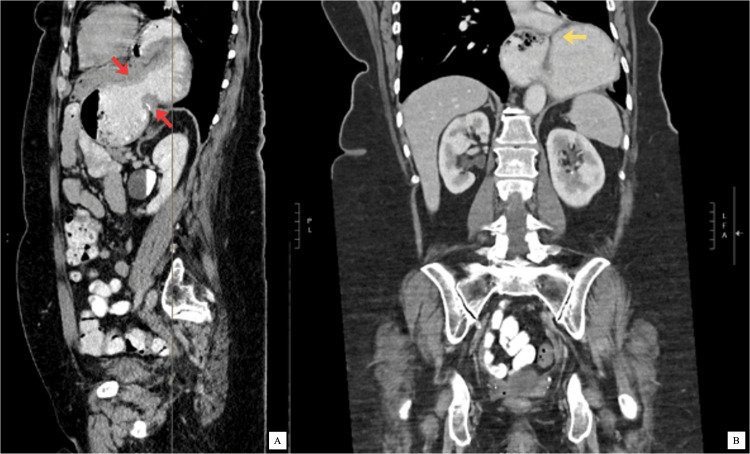
Case 1: Sagittal (A) and coronal (B) views of a CT abdomen/pelvis demonstrating a large hiatal hernia with changes consistent with the patient's history of fundoplication. Red arrows (A): Both point to the herniated stomach above the diaphragm - the upper arrow is on the fundoplication wrap (the heterogeneous/mottled density from the wrapped fundus), while the lower arrow is on the herniated gastric body/hernia sac itself. Yellow arrow (B): Same structure, coronal view - pointing at the wrap/herniated stomach sitting above the diaphragm on the left side. CT: computed tomography

**Figure 2:**
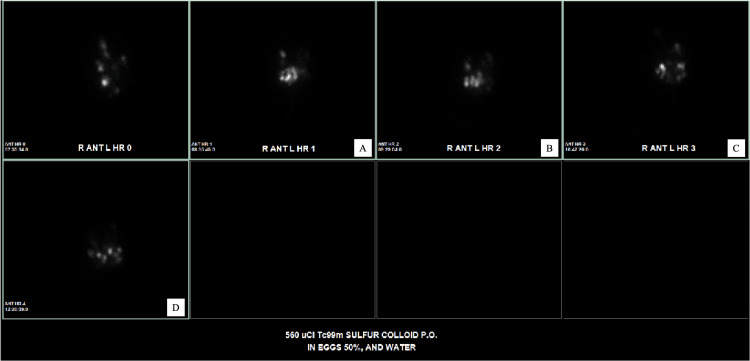
Case 1: Nuclear medicine gastric emptying study. The nuclear medicine gastric emptying study reveals that > 95% of the contents remained in the stomach at one hour (A), 95% remained at two hours (B), 75% remained at three hours (C), and 70% remained at four hours (D). Slow and incomplete clearance of radiotracer from the stomach after delayed four-hour static images is consistent with gastroparesis. Only the boxes labeled are relevant.

A 70-year-old woman with a past medical history significant for multiple sclerosis (MS), gastroesophageal reflux disease (GERD), eosinophilic esophagitis, and hiatal hernia status-post Nissen fundoplication presented for evaluation and management of gastroparesis. She initially sought guidance from her primary care provider, during which she reported a 40-pound unintentional weight loss, anorexia, and worsening indigestion. CT abdomen/pelvis revealed a stable large hiatal hernia with changes consistent with the patient’s past surgical history (Figure 1). She was advised to continue pantoprazole daily in the morning, start famotidine at night, and was referred to gastroenterology. Upper GI endoscopy revealed an irregular Z-line, gastritis (biopsied), food in the lower third of the esophagus (removed), a normal duodenum, and a large amount of food residue in the stomach (>500 mL removed). A nuclear medicine gastric emptying study revealed slow and incomplete clearance of radiotracer from the stomach after delayed four-hour static images consistent with gastroparesis (Figure 2).

Five months later, she presented as an outpatient to General Surgery with concerns of persistent nausea and vomiting, continued unintentional weight loss, epigastric abdominal pain, and constipation. The assessment at the time was idiopathic gastroparesis versus post-surgical gastroparesis, with discussion that current symptoms may be related to MS versus hiatal hernia, and the decision was made to proceed with gastric peroral endoscopic myotomy (G-POEM). One month later, admission workup revealed epigastric abdominal tenderness but an otherwise unremarkable physical examination. The patient was taken to the operating room for planned G-POEM; however, intraoperative esophagogastroduodenoscopy (EGD) revealed Los Angeles (LA) grade D esophagitis, large retained gastric food/fluid burden, and a patent pylorus, prompting recognition that the underlying pathology was a gastric volvulus rather than a pyloric dysfunction. The decision was then made to abort G-POEM, after which emergency consent was obtained, and the transition to definitive operative management began. The patient underwent robotic hiatal hernia repair with mesh, reduction of a mesenteroaxial gastric volvulus, Collis gastroplasty, diaphragmatic relaxing incision, and gastropexy. Findings included a large type III paraesophageal hernia, dense adhesions, and prior surgical changes from a previous Nissen fundoplication, which complicated the dissection and reduction. The patient was admitted postoperatively. Overall, she had an uneventful recovery and was discharged home six days later with close outpatient surgical follow-up. At the time of discharge, the patient was tolerating her diet, independently ambulating, and was hemodynamically stable. Since then, she has been seen in the clinic and is gaining weight, denies nausea or vomiting, and is consuming a regular diet.

Case 2: Roux-en-O after gastric bypass

A 53-year-old woman with class II obesity (body mass index (BMI): 36.9 kg/m^2^) underwent RYGB and hiatal hernia repair. Her preoperative weight was approximately 262 pounds (BMI: 47.9 kg/m^2^). Relevant medical and surgical history included irritable bowel syndrome with constipation (IBS-C), GERD, colonic inertia status-post left hemicolectomy, generalized anxiety disorder/major depressive disorder (GAD/MDD), bipolar disorder, migraines, fibromyalgia, obstructive sleep apnea (OSA), and asthma. Following the RYGB, the patient had prolonged and repeated hospitalizations secondary to ileus, small-bowel obstruction, and incarcerated ventral hernia requiring exploratory laparotomy with small bowel resection and lysis of adhesions. No abnormality of the RYGB was noted at the exploratory laparotomy. She subsequently underwent cholecystectomy for acute cholecystitis due to concern that it was contributing to her symptoms.

**Figure 3:**
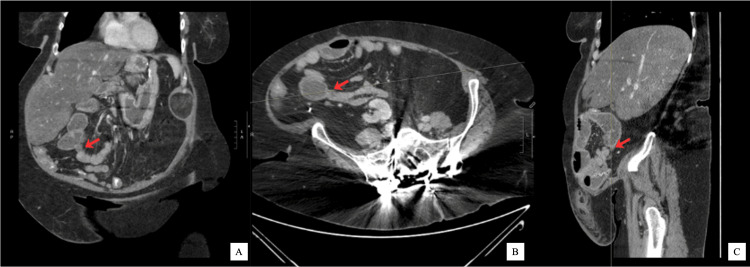
Case 2: Coronal (A), axial (B), and sagittal (C) views of a CT abdomen/pelvis demonstrating dilated loops of small bowel with a transition point at the surgical anastomosis, as well as postoperative changes and fat stranding in the abdominal wall. This CT abdomen/pelvis shows dilated fluid-filled small bowel extending from the gastrojejunostomy site with a transition point in the right lower quadrant (red arrows) at the surgical anastomosis. CT: computed tomography

Three months following the RYGB, the patient presented with persistent abdominal pain, nausea, vomiting, inability to tolerate oral intake, and worsening gastroesophageal reflux with bilious regurgitation partially responsive to metoclopramide and ondansetron. These symptoms had been present and persistent since her index surgery. Vital signs were stable, and physical examination was without signs of peritonitis or other acute findings. Initial workup revealed leukocytosis with a white blood cell count of 14.34 x 10^3^/µL (reference range: 4.60-10.20 x 10^3^/µL), hypoalbuminemia with a serum albumin level of 3.4 g/dL (reference range: 3.5-5.0 g/dL), and anemia of chronic inflammation with a hemoglobin level of 11.5 g/dL (reference range: 12.2-16.2 g/dL). CT abdomen/pelvis demonstrated dilated loops of small bowel with a transition point at the surgical anastomosis, as well as postoperative changes and fat stranding in the abdominal wall (Figure 3).

It was difficult to determine how many of the patient’s symptoms were present before the operative bypass. Given a history of retained food on endoscopy, it was posited that there was an element of gastroparesis. With the presumed diagnosis of gastroparesis, two potential avenues for improvement were discussed with the patient. The first option was placing a feeding tube in the remnant stomach, which could aid in the decision whether she needs to have the removal of her remnant stomach. The second option was a gastric neurostimulator, which would be effective in relieving her nausea and vomiting despite her bypass anatomy. We also discussed the possibility of reversal of her gastric bypass. At that time, review of the patient's CT scan revealed some dilation of her gastric remnant and BP limb, with examination of the mesentery suggesting that there was a small internal hernia present, which could have also explained her postoperative symptoms. The plan was made to place a gastrostomy tube in her remnant stomach as well as a gastric neurostimulator, and to evaluate at the time of surgery for a potential hernia.

The patient subsequently underwent robotic-assisted diagnostic laparoscopy converted to open laparotomy, reversal of gastric bypass, pyloroplasty, small bowel resection (x3), lysis of adhesions, and resection of an incidentally discovered large diaphragmatic lipomatous mass. Intraoperative findings consisted of abnormal bypass anatomy and adhesions causing impaired drainage. The patient’s BP limb was anastomosed to her pouch, and the Roux limb was attached to this U-shaped BP limb (Roux-en-O configuration with twisting of the limbs). Due to this, the patient did not have appropriate drainage for her bypass, which likely led to her nausea and vomiting. The gastric bypass was reversed, and the stomach was reconnected with a pyloroplasty performed due to concern for preoperative gastroparesis and poor pyloric function after reversal. Postoperatively, she was managed conservatively with a nasogastric tube (NGT) due to concern for postoperative ileus. She was discharged home after 11 days. She was then seen in the clinic and reported that her symptoms of nausea, vomiting, and GERD were completely resolved.

## Discussion

Before confirming the diagnosis of a motility disorder, such as gastroparesis, systematic exclusion of mechanical obstruction must occur. By definition, a gastroparesis diagnosis requires delayed gastric emptying in the setting of the absence of gastric outlet obstruction, usually confirmed by upper endoscopy [7].

Case 1 illustrates how chronic gastric volvulus can present over months, mimicking the slow and indolent course of gastroparesis, rather than the acute obstructive presentation typically described in the current literature. This case also illustrates the limitations of standard diagnostic testing for gastroparesis. This patient had delayed gastric emptying confirmed by scintigraphy and G-POEM scheduled for presumed gastroparesis. However, intraoperative endoscopy revealed a patent pylorus with retained gastric contents, suggesting obstruction not at the level of the pylorus, but at the stomach. Delayed emptying may be a result of gastroparesis or structural causes such as gastric volvulus, but gold standard imaging (scintigraphic gastric emptying) cannot distinguish between mechanical obstruction and functional delay [8].

Case 2 describes a patient with intractable abdominal pain, nausea, vomiting, inability to tolerate oral intake, and worsening gastroesophageal reflux with bilious regurgitation following RYGB. The diagnosis of Roux-en-O was only confirmed with surgical exploration. Importantly, the patient had a history of psychiatric illness (GAD/MDD). Psychiatric comorbidities may impact symptom presentation, management, and workflow. There may be value in consulting with mental health teams for more insight into these types of cases. This case demonstrates not only the diagnostic challenges following bariatric surgery, but also that surgical exploration may be necessary to definitively diagnose when imaging is non-diagnostic. Nausea and vomiting after RYGB can stem from different etiologies, including marginal ulcers, strictures, internal hernia, and it is even the most common postoperative complication after surgery [9]. Due to altered anatomy following gastric bypass, gastric emptying studies cannot be performed, thus eliminating a crucial diagnostic tool [10].

There are a few clinical lessons to take away from this case series. Maintain a high degree of suspicion for underlying structural abnormalities in patients who present with gastroparesis-like symptoms, especially in atypical presentations, persistence of symptoms, or patients with previous bariatric/foregut surgery. Recognize that standard testing has limitations: gastric emptying studies can confirm delayed emptying but cannot distinguish the mechanism, endoscopy may miss intermittent or chronic volvulus, and CT imaging for internal hernia or surgical misconstructions has imperfect sensitivity. Similarly, when imaging is non-diagnostic, or symptoms persist or are refractory despite treatment, consider surgical exploration for definitive diagnosis.

## Conclusions

Nausea, vomiting, and delayed gastric emptying may not always be synonymous with gastroparesis. Gastroparesis is a diagnosis of exclusion that requires systematic evaluation to rule out mechanical and structural causes. This case series demonstrates that structural abnormalities such as gastric volvulus and the Roux-en-O configuration can present with chronic, indolent symptoms that closely mimic gastroparesis over months, rather than acute obstructive presentations typically described in the literature. Patients with refractory symptoms, atypical presentations, or previous foregut/bariatric surgery pose unique diagnostic challenges and require heightened clinical suspicion, as delayed diagnosis can lead to life-threatening complications, including gastric ischemia, perforation, or progressive obstruction. When gastroparesis-like symptoms do not fit the typical pattern, consider that it may not be gastroparesis at all.
